# Epithelial Cell Transforming Sequence 2 in Human Oral Cancer

**DOI:** 10.1371/journal.pone.0014082

**Published:** 2010-11-29

**Authors:** Manabu Iyoda, Atsushi Kasamatsu, Takashi Ishigami, Dai Nakashima, Yosuke Endo-Sakamoto, Katsunori Ogawara, Masashi Shiiba, Hideki Tanzawa, Katsuhiro Uzawa

**Affiliations:** 1 Department of Clinical Molecular Biology, Graduate School of Medicine, Chiba University, Chiba, Japan; 2 Division of Dentistry and Oral-Maxillofacial Surgery, Chiba University Hospital, Chiba, Japan; Deutsches Krebsforschungszentrum, Germany

## Abstract

**Background:**

Epithelial cell transforming sequence 2 (ECT2) is a guanine nucleotide exchange factor for Rho family GTPase, which has been implicated in the malignant phenotype of human cancers. Little is known about the effect of a high level of ECT2 in regulating oral cancer cell behavior. In this study, we investigated the involvement of ECT2 in oral squamous cell carcinoma (OSCC).

**Methodology/Principal Findings:**

We analyzed ECT2 expression in OSCC-derived cell lines and primary OSCCs compared with matched normal tissue (n = 96) by quantitative reverse transcriptase-polymerase chain reaction, Western blot, and immunohistochemistry. We then evaluated the correlation between the ECT2 expression status in primary OSCCs and the clinicopathological features. ECT2 expression was significantly up-regulated in OSCCs *in vitro* and *in vivo* (*p*<0.05). Among the clinical variables analyzed, higher ECT2 expression also was associated with the TNM stage grading (*p*<0.05). When we performed functional analyses of ECT2 in OSCC-derived cells using the shRNA system, the cellular proliferation of the ECT2 knockdown cells decreased significantly compared with the control cells (*p*<0.05). Cell cycle analysis by flow cytometry showed arrest of cell cycle progression at the G1 phase in the ECT2 knockdown cells. We also found up-regulation of the Cip/Kip family of the cyclin-dependent kinase inhibitors, p21^cip1^ and p27^kip1^, and down-regulation of cyclin D1, cyclin E, and CDK4. These data suggested that the elevated Cip/Kip family induced inhibition of the cyclin D1-CDK complex activity leading to cell cycle arrest at the G1 phase.

**Conclusions/Significance:**

Our results proposed for the first time that ECT2 is an indicator of cellular proliferation in OSCCs and that ECT2 might be a potential therapeutic target for the development of new treatments for OSCCs.

## Introduction

Oral squamous cell carcinoma (OSCC) is a major cause of morbidity and mortality globally, accounting for 275,000 new cases and more than 120,000 deaths annually [1]. Many risk factors have been identified, including tobacco and alcohol use [2], [3], [4]. However, some patients develop OSCC without risk factors, suggesting that host susceptibility plays an important role. Molecular changes in a number of oncogenes and tumor suppressor genes associated with the development of OSCC could be important clues to preventing this disease [4], [5].

Microarray technology has been helpful for analyzing changes in thousands of genes and identifying significant patterns. We previously reported gene expression profiling of OSCC to identify cancer-related genes [6]. Among the genes, epithelial cell transforming sequence 2 (ECT2) was significantly up-regulated in OSCC. ECT2 is a guanine nucleotide exchange factor (GEF) for Rho family GTPase related to cytokinesis [7], [8], [9], [10], [11]. GEFs catalyze the exchange of GDP for GTP, thereby activating the Rho GTPases in signal transduction. ECT2 expression is dynamically controlled throughout the cell cycle. Upon breakdown of the nuclear envelope during mitosis, ECT2 is dispersed throughout the cytoplasm, then ECT2 becomes localized to the mitotic spindles during metaphase, the cleavage furrow during telophase, and the mid-body at the end of cytokinesis [8]. The Rho GTPases have been implicated in the malignant phenotype of human cancers as a result of their participation in aberrant signaling in tumor cells [12], [13], [14], [15], [16], [17].

In the current study, ECT2 was frequently overexpressed in OSCC-derived cell lines and primary OSCCs. In addition, a shRNA experiment showed that ECT2 down-regulation resulted in decreased cellular proliferation by cell cycle arrest of the G1 phase. Therefore, we suggested that ECT2 might be a biomarker of proliferation and potential therapeutic target for OSCCs.

## Results

### Evaluation of *ECT2* mRNA expression in OSCC-derived cell lines

**Figure 1:**
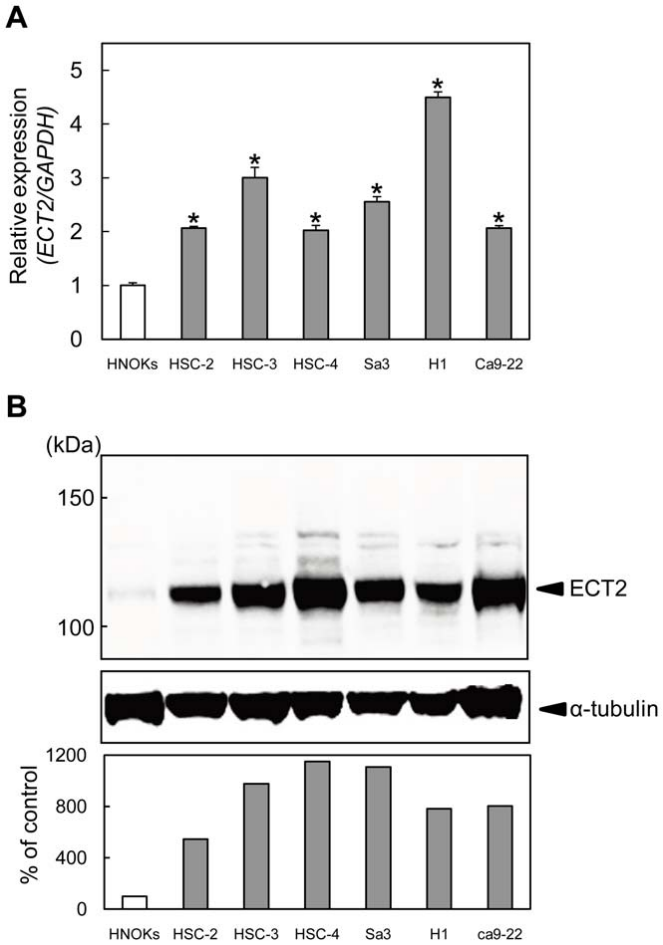
ECT2 exprssion in OSCC-derived cell lines. (**A**) Quantification of *ECT2* mRNA levels in OSCC-derived cell lines by qRT-PCR analysis. To determine mRNA expression of *ECT2* in Oral cancer, we performed qRT-PCR analysis using six OSCC-derived cell lines (HSC-2, HSC-3, HSC-4, H1, Ca9-22, and Sa3) and HNOKs. Significant up-regulation of *ECT2* mRNA is seen in six OSCC-derived cell lines compared with that in the HNOKs. Data are expressed as the means ± SEM of values from three assays (*p<0.05; Mann-Whitney *U* test). (**B**) Western blot analysis of ECT2 protein in the OSCC-derived cell lines and HNOKs. To investigate protein expression of ECT2 in Oral cancer, we performed Western blot analysis using six OSCC-derived cell lines (HSC-2, HSC-3, HSC-4, H1, Ca9-22, and Sa3) and HNOKs. ECT2 protein expression is up-regulated in OSCC-derived cell lines compared with HNOKs. Densitometric ECT2 protein data are normalized to α-tubulin protein levels. The values are expressed as a percentage of the HNOKs.

To investigate mRNA expression of *ECT2* identified as a cancer-related gene by our microarray analysis [6], we performed quantitative reverse transcriptase PCR (qRT-PCR) analysis using six OSCC-derived cell lines (HSC-2, HSC-3, HSC-4, H1, Ca9-22, and Sa3) and human normal oral keratinocytes (HNOKs). mRNA expression levels were normalized to GAPDH. *ECT2* mRNA was significantly up-regulated in all OSCC cell lines compared with the HNOKs (Figure 1A, *p<0.05).

### Evaluation of ECT2 protein expression in OSCC-derived cell lines

We performed Western blot analysis to investigate ECT2 protein expression status in the OSCC-derived cell lines and the HNOKs (Figure 1B). The molecular weight of the ECT2 was 112 kDa. A significant increase in ECT2 protein expression was observed in all OSCC cell lines compared with the HNOKs. Expression analysis indicated that both transcription and translation products of this molecule were highly expressed in OSCC-derived cell lines.

### Evaluation of ECT2 expression in primary OSCCs

**Figure 2:**
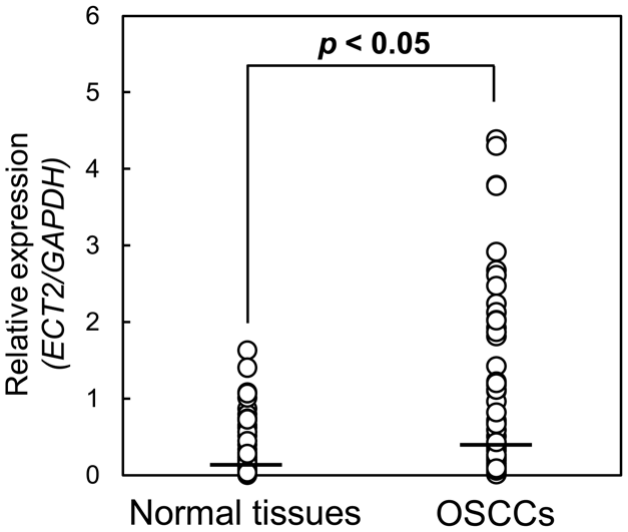
Comparison of *ECT2* mRNA expression levels between primary OSCCs and matched normal oral tissues. To investigate the *ECT2* mRNA expression levels in primary OSCCs and paired normal oral tissues from 96 patients, we performed qRT-PCR analysis. The relative mRNA expression levels in primary OSCCs and the matched oral tissues (n = 96) range from 0.005 to 4.39 (median, 0.289) and 0.003 to 1.632 (median, 0.081), respectively. *ECT2* mRNA expression was up-regulated in 75 (78%) of 96 primary OSCCs compared with the matched normal oral tissues. Significantly higher *ECT2* mRNA expression was observed in primary OSCCs than matched normal oral tissues (*P*<0.05; Mann-Whitney *U* test).

We measured the *ECT2* mRNA expression levels in primary OSCCs and paired normal oral tissues from 96 patients. Similar to the data from the OSCC-derived cell lines, qRT-PCR analysis showed that *ECT2* mRNA expression was up-regulated in 75 (78%) of 96 primary OSCCs compared with the matched normal oral tissues. The relative mRNA expression levels in the normal oral tissues and primary OSCCs ranged from 0.003 to 1.632 (median, 0.081) and 0.005 to 4.39 (median, 0.289), respectively (Figure 2, *p*<0.05).

**Figure 3:**
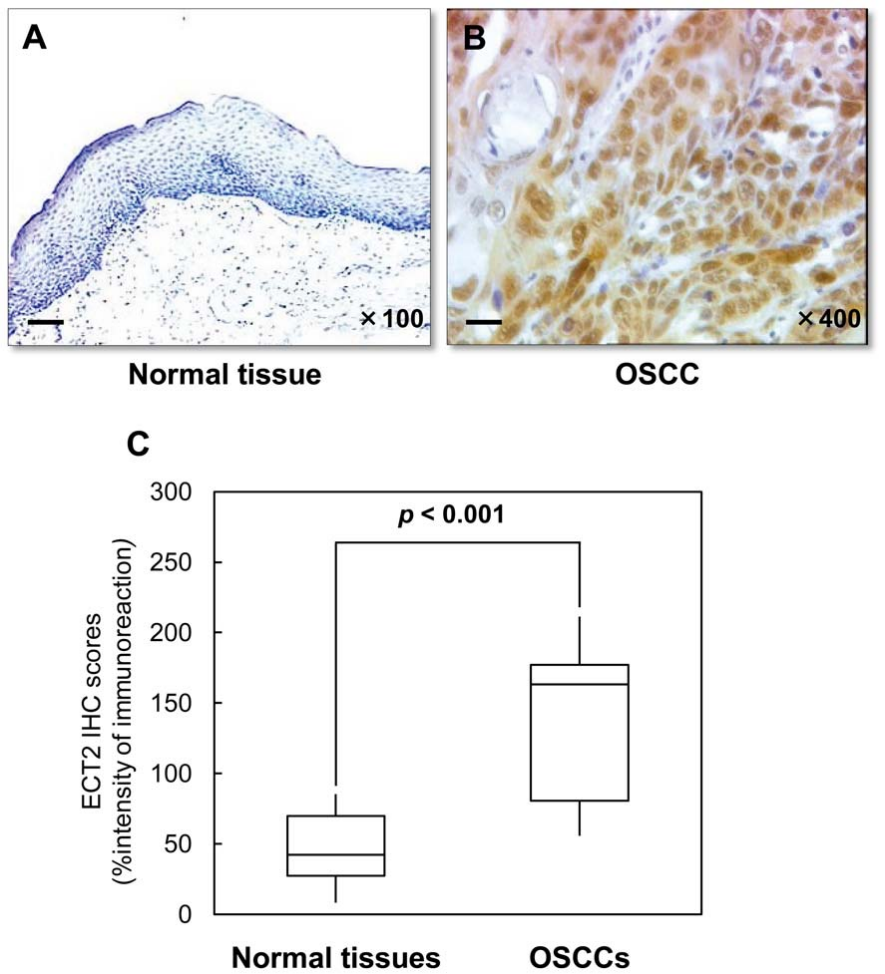
Evaluation of ECT2 protein expression in primary OSCCs. (**A**, **B**) Representative IHC results of ECT2 in normal oral tissue and primary OSCC. (**A**) Normal oral tissue has no ECT2 protein expression. Original magnification, ×100. Scale bars, 50 µm. (**B**) ECT2-positive cases of OSCC. Positive immunoreaction for ECT2 is detected in the nucleus and cytoplasm. Original magnification, ×400. Scale bars, 10 µm. (**C**) State of ECT2 protein expression in nomal oral tissue and primary OSCC. To investigate protein expression of ECT2 in primary OSCCs, we carried out IHC. The ECT2 IHC scores are calculated as follows: IHC score = 1×(number of weak stained cells in the field)+2×(number of moderately stained cells in the field)+3×(number of intensely stained cells in the field). The ECT2 IHC scores for OSCCs and normal oral tissues range from 55.67 to 211.33 (median, 163.33) and 8.33 to 85.33 (median, 44.00), respectively. The ECT2 protein expression level in OSCCs is significantly higher than that in normal oral tissues (*p*<0.001; Mann-Whitney *U* test).

We then analyzed ECT2 protein expression by immunohistochemistry (IHC). Representative IHC results for ECT2 protein in normal oral tissue and primary OSCC are shown in Figure 3A and B. Positive immunoreaction for ECT2 was detected in the nucleus and cytoplasm. Strong ECT2 immunoreactions were detected in OSCCs, whereas normal oral tissues showed negative immunostaining. The ECT2 IHC scores of normal oral tissues and OSCCs ranged from 8.33 to 85.33 (median, 44.00) and 55.67 to 211.33 (median, 163.33), respectively. The ECT2 IHC scores in primary OSCCs were significantly higher than those in normal tissues (Figure 3C, *p*<0.001). The correlations between the clinicopathologic characteristics of the patients with OSCC and the status of ECT2 protein expression using the IHC scoring system are shown in Table 1. Among the clinical classifications, ECT2-positive OSCCs were correlated with tumor size (*p* = 0.043) and TNM staging of OSCC (*p* = 0.044) (Table 1).

**Table 1:** Correlation between ECT2 expression and clinical classification in OSCCs.

		Results of immunostaining				
		No. patients/%				
Clinical classification	Total	ECT2(−)		ECT2(+)		*p* value
Age at surgery (years)						
<60	28	7	25%	21	75%	0.136
60–70	27	4	15%	23	85%	
70>	41	15	37%	26	63%	
Gender						
Male	67	19	28%	48	72%	0.669
Female	29	7	24%	22	76%	
T-primary tumor size						
T1	13	5	38%	8	62%	0.043*
T2	31	11	35%	20	65%	
T3	24	5	21%	19	79%	
T4	28	5	18%	23	82%	
T1+T2	43	16	37%	27	63%	0.044*
T3+T4	53	10	19%	43	81%	
N-regional lymph node metastasis						
N positive	43	15	35%	28	65%	0.121
N negative	53	11	21%	42	79%	
Stage						
I	9	5	56%	4	44%	0.248
II	15	6	40%	9	60%	
III	16	2	13%	14	87%	
IV	56	13	23%	43	77%	
I+II	24	11	46%	13	54%	0.017*
III+IV	72	15	21%	57	79%	
Histopathologic type						
Well differentiated	64	20	31%	44	69%	0.275
Moderately differentiated	28	6	21%	22	79%	
Poorly differentiated	4	0	0%	4	100%	
Tumor site						
Gingiva	23	3	13%	20	87%	0.807
Tongue	54	19	35%	35	65%	
Buccal mucosa	10	3	30%	7	70%	
Oral floor	7	1	14%	6	86%	
Oropharyngeal isthmus	1	0	0%	1	100%	
Soft palate	1	0	0%	1	100%	

*p<0.05. ECT2(+), ECT2-positive case; ECT2(−), ECT2-negative case.

### Establishment of ECT2 knockdown cells

**Figure 4:**
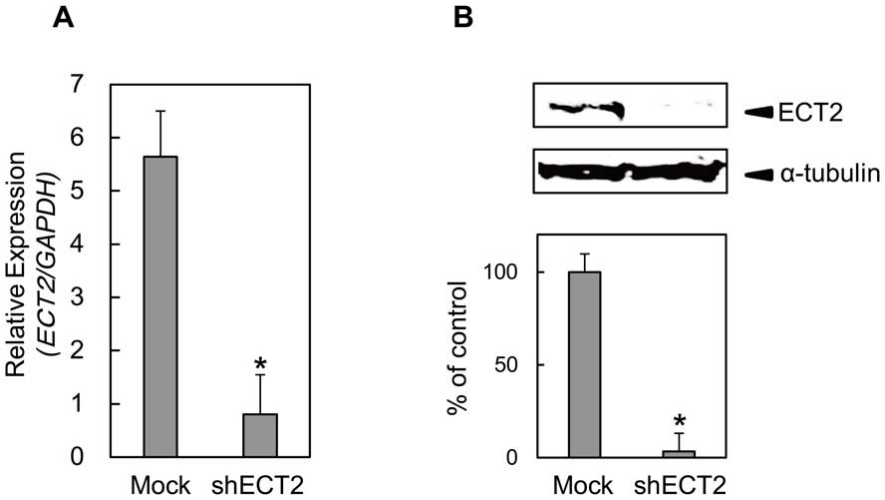
Expression ECT2 in shECT2-transfected cells. To obtain stable ECT2 knockdown transfectants, we performed transfection of the ECT2 shRNA (shECT2) and the control shRNA (Mock) in OSCC cell lines (Sa3 and H1). We performed qRT-PCR and Western blot analyses to investigate ECT2 mRNA and protein expression in shECT2-transfected cells. (**A**) Expression of *ECT2* mRNA in shECT2- and Mock-transfected Sa3 cells. (**B**) Western blot analysis of ECT2 protein in shECT2- and Mock-transfected cells. The ECT2 mRNA and proteins are significantly down-regulated in shECT2-transfected cells.

To obtain stable ECT2 knockdown transfectants, we used the ECT2 shRNA (shECT2) plasmid and the control shRNA (Mock) plasmid. To assess ECT2 mRNA and protein expression in shECT2-transfected cells, we performed qRT-PCR and Western blot analyses. Figure 4A shows that the *ECT2* mRNA expression in shECT2-transfected cells was significantly lower than in Mock-transfected cells. ECT2 protein levels in shECT2-transfected cells also decreased markedly compared with Mock-transfected cells (Figure 4B). ECT2 protein expression levels were consistent with the mRNA expression in the transfectants.

### Reduced cellular growth in ECT2 knockdown cells

**Figure 5:**
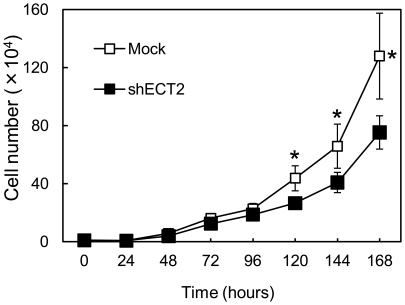
Proliferation of shECT2-transfected cells. To determine the effect of shECT2 on cellular proliferation, shECT2- and Mock-transfected cells were seeded in 6-well plates at a density of 1×10^4^ viable cells per well. shECT2- and Mock-transfected cells counted on 7 consecutive days. The growth of shECT2-transfected cells is significantly inhibited compared with the Mock-transfected cells after 7 days. The results are expressed as the means ± SEM of values from three assays. The asterisks indicate significant differences between the Mock- and shECT2-transfected cells (*p*<0.01; Mann-Whitney *U* test).

To investigate the antiproliferative effects in shECT2-transfected cells, cellular growth was monitored for 7 days. The shECT2-transfected cells showed a significant decrease in cellular growth compared with Mock-transfected cells (Figure 5).

### Knockdown of ECT2 promotes cell cycle arrest

**Figure 6:**
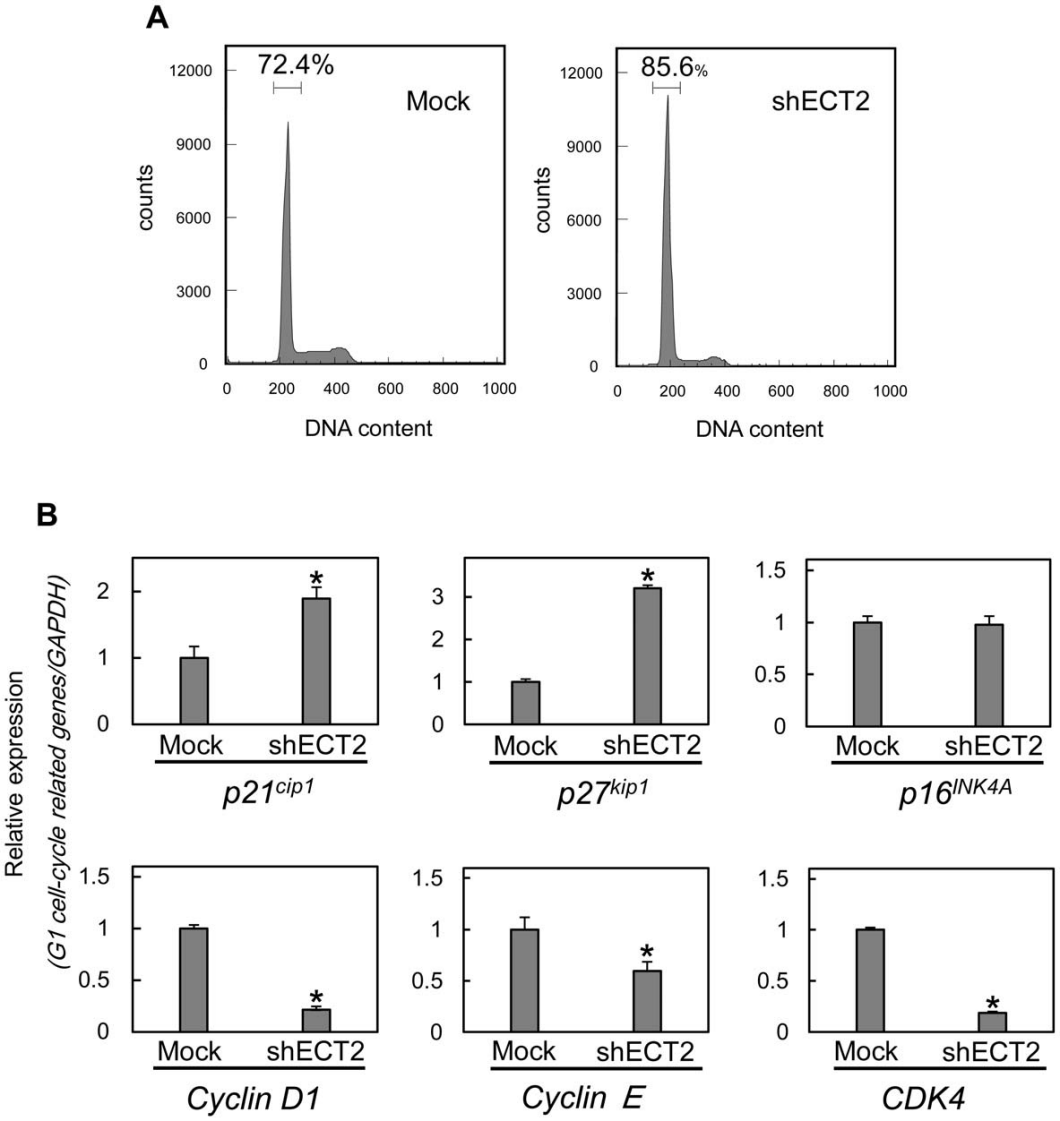
shECT2 promotes G1 arrest. To investigate cell cycle progression, we analyzed Flow cytometric determination of DNA content by a FACScalibur in the G0–G1, S, and, G2–M phases. We then determined the expression level of cyclin-dependent kinase inhibitors (p16^INK4A^, p21^cip1^, and p27^kip1^), cyclin D1, cyclin E, and CDK4 to identify the mechanism by which ECT2 blocks G1 progression. (**A**) Flow cytometric analysis was performed to investigate cell cycle in shECT2- and Mock-transfected cells. The number of cells in the G1 has increased markedly in the ECT2 knockdown cells. (**B**) qRT-PCR was performed to investigate mRNA levels of cell cycle related genes. PCR shows up-regulation of *p21^cip1^* and *p27^kip1^* and down-regulation of *cyclin D1*, *cyclin E*, *and CDK4*. Data are expressed as the means ± SEM of values from three assays (*p<0.05; Mann-Whitney *U* test).

To investigate the mechanism by which ECT2 is related to cell cycle progression, we performed FACS analysis of shECT2-transfected cells. The percentage of the G1 phase in shECT2-transfected cells was significantly higher than in Mock-transfected cells (Figure 6A, *p*<0.05), suggesting that down-regulation of ECT2 inhibited cellular proliferation by induction of G1 arrest. To identify the mechanism by which ECT2 blocks G1 progression, we assessed the expression level of cyclin-dependent kinase inhibitors (p16^INK4A^, p21^cip1^, p27^kip1^), cyclin D1, cyclin E, and CDK4 (Figure 6B). PCR data showed up-regulation of *p21^cip1^* and *p27^kip1^* and down-regulation of *cyclin D1*, *cyclin E*, *and CDK4* in shECT2-transfected cells.

## Discussion

Our previous microarray data [6] showed significant up-regulation of *ECT2* in OSCC-derived cell lines. In the present study, ECT2 mRNA and protein were highly expressed *in vitro* and *in vivo* in OSCC. Regional copy number of 3q26 increases in several cancers, such as head and neck, lung, and cervix [18], [19]. This region has cancer-related genes (PRKC1 and SOX2) as well as ECT2. Therefore, genomic unbalance would be the reason of ECT2 overexpression in OSCC. The ECT2 protein expression levels in primary OSCCs were correlated with the TNM stage grading (Table 1) (*p*<0.05). These results suggested that ECT2 has an important role in OSCC development and progression. However, little is known about the mechanism of ECT2 in OSCC progression. To determine whether ECT2 function is relevant to OSCC progression, we performed the shECT2 experiment and found that cellular proliferation decreased significantly as a result of cell cycle arrest at the G1 phase in ECT2 knockdown cells with up-regulation of p21^cip1^ and p27^kip1^ and down-regulation of cyclin D1, cyclin E, and CDK4, indicating that ECT2 function is related closely to OSCC progression.

GEFs, including ECT2, catalyze the exchange of GDP for GTP, thereby activating the Rho GTPases in signal transduction [7], [8], [9], [10], [11]. Activated Rho GTPases bind to and activate several downstream effectors, leading to multiple biologic processes, such as cellular size, cell cycle progression, apoptosis, survival, morphology, cellular polarity, cellular adhesion, and membrane trafficking [20], [21]. Up-regulation of Rho GTPase activity, often associated with tumorigenesis [22], has been detected in several human tumors, including pancreatic cancer, breast cancer, melanoma, lung cancer, colorectal cancer, and gastric cancer [12], [23]. On the other hand, Rho GTPases play a major role in promoting G1-S progression through modulation of cyclin and cyclin dependent kinase inhibitors (CDKIs) [24], [25]. Yamamoto et al. reported that when Rho GTPase was inhibited by the *Clostridium botulinum* C3 toxin or a dominant negative mutant, G1-S cell cycle progression was significantly impaired [26]. The impaired activation of GTPases is associated with constitutively elevated levels of p21^cip1^ and p27^kip1^, causing cells to accumulate in the G1 phase [27], [28], [29], [30], [31], [32]. We speculated that ECT2 knockdown leads to impaired activation of Rho GTPase, and consistent with that, we found not only up-regulation of the Cip/Kip family (p21^cip1^ and p27^kip1^) but also down-regulation of cyclin D1, cyclin E, and CDK4, leading to cell cycle arrest at the G1 phase, in ECT2 knockdown cells.

Cyclin D1, cyclin E, and CDK4 are also a critical regulator of G1 progression and G1-S transition. Inhibition of cyclin D1, cyclin E, and CDK4 expression blocks G1-S transition in the cell cycle [33], [34], [35], [36]. Cyclins D1–D3 and E families and their respective kinase partners, CDK4/6 and CDK2, are responsible for regulating the transition from G1 to S phase. The activities of the cyclin-CDK complexes are modulated by two types of CDKIs, Cip/Kip (p21^Cip1^, p27^Kip1^, and p57^Kip2^) and the INK4 (p15^INK4B^, p16^INK4A^, p18^INK4C^, and p19^INK4D^) families, both of which regulate cell cycle progression [37]. Members of the Cip/Kip family bind to cyclin-CDK complexes and inhibit their activities, which leads to reduced phosphorylated retinoblastoma protein and G1 cell cycle arrest.

In conclusion, our results indicated that ECT2 is overexpressed frequently in OSCC. Furthermore, ECT2 knockdown inhibited cellular proliferation *in vitro* by arresting cell cycle progression at the G1 phase by modulating expression of cell cycle-related molecules, which ultimately leads to inhibition of cyclin D1-CDK complex activity. These data suggested that ECT2 plays an important role in OSCC cell proliferation. ECT2 expression is likely to be a biomarker of proliferation and a potential therapeutic target for development of anticancer drugs in primary OSCCs.

## Materials and Methods

### Ethics Statement

All patients provided informed consent for a protocol reviewed and approved by the institutional review board of Chiba University. The written informed consents were obtained from all patients.

### OSCC-derived cell lines and tissue specimens

HSC-2, HSC-3, HSC-4, and Ca9-22 cell lines, derived from human OSCCs, were purchased from the Human Science Research Resources Bank (Osaka, Japan). H1 and Sa3 cell lines were kindly provided by Dr. S. Fujita at Wakayama Medical University (Wakayama, Japan). Primary cultured HNOKs were obtained from three healthy donors [38], [39]. All cells were grown in Dulbecco's modified Eagle medium/F-12 HAM (Sigma-Aldrich Co, St. Louis, MO) supplemented with 10% fetal bovine serum (Sigma) and 50 units/ml penicillin and streptomycin (Sigma).

Tissue samples from 96 unrelated Japanese patients with primary OSCC who were treated at the Chiba University Hospital were obtained during surgical resection. The resected tissues were divided into two parts, one of which was frozen immediately and stored at −80°C until RNA isolation, and the second of which was fixed in 10% buffered formaldehyde solution for pathologic diagnosis and IHC. Histopathologic analysis of the tissues was performed according to the World Health Organization criteria by the Department of Pathology, Chiba University Hospital. Clinicopathologic staging was determined by the TNM classification of the International Union against Cancer. All patients had OSCC that was histologically confirmed, and tumor samples were checked to ensure that tumor tissue was present in more than 90% of the specimen.

### Preparation of cDNA

Total RNA was isolated using Trizol Reagent (Invitrogen, Carlsbad, CA) according to the manufacturer's instructions. cDNA was generated from 5 µg of total RNA using Ready-To-Go You-Prime First-Strand Beads (GE Healthcare, Buckinghamshire, UK) and oligo (dT) primer (Sigma Genosys, Ishikari, Japan), according to the manufacturer's instructions.

### mRNA expression analysis

Real-time qRT-PCR was performed to evaluate the expression levels of target genes (*ECT2*, *p16^INK4A^*, *p21^cip1^*, *p27^kip1^*, *cyclin D1*, *cyclin E*, and *CDK4*) in OSCC-derived cells and primary OSCCs. qRT-PCR was carried out with one method using a LightCycler FastStart DNA Master SYBR Green 1 Kit (Roche Diagnostics GmbH, Mannheim, Germany). The following primers were used: ECT2, forward 5′-ATTTTCATGTCGCCCGTTGT-3′ and reverse 5′-CCCATGTGATGGACCAATGTC-3′; p16^INK4A^, forward 5′-CAGACATCCCCGATTGAAAGAAC-3′ and reverse 5′-GGTAGTGGGGGAAGGCATATATCT-3′; p21^cip1^, forward 5′-CCCAGTTCATTGCACTTTGATTAGC-3′and reverse 5′-CAGTCTAGGTGGAGAAACGGGAAC-3′; p27^kip1^, forward 5′-CCGGCTAACTCTGAGGACAC-3′and reverse 5′-AGAAGAATCGTCGGTTGCAG-3′; cyclin D1, forward 5′-GCATGTTCGTGGCCTCTAAGA-3′and reverse 5′-CGGTGTAGATGCACAGCTTCTC-3′; cyclin E, forward 5′-TTCTTGAGCAACACCCTCTTCTGCAGCC-3′and reverse 5′-TCGCCATATACCGGTCAAAGAAATCTTGTGCC-3′; CDK4, forward 5′-TGCAACACCTGTGGACATGTG-3′and reverse 5′-ATTTTGCCCAACTGGTCGG-3′. Amplified products were analyzed by 3% agarose gel electrophoresis to ascertain size and purity. The PCR reactions using the LightCycler apparatus were performed in a final volume of 20 µl of a reaction mixture consisting of 2 µl of FirstStart DNA Master SYBR Green I mix, 3 mM MgCl_2_, and l µM of the primers, according to the manufacturer's instructions. The reaction mixture was loaded into glass capillary tubes and subjected to an initial denaturation at 95°C for 10 min, followed by 45 rounds of amplification at 95°C (10 sec) for denaturation, 62°C (10 sec) for annealing, and 72°C (10 sec) for extension, with a temperature slope of 20°C/sec. The transcript amounts for the target genes were estimated from the respective standard curves and normalized to the glyceraldehyde-3-phosphate dehydrogenase (GAPDH) (forward 5′-CATCTCTGCCCCCTCTGCTGA-3′and reverse 5′-GGATGACCTTGCCCACAGCCT-3′) transcript amount determined in corresponding samples.

### Protein extraction

The cells were washed twice with cold phosphate-buffered saline (PBS) and centrifuged briefly. The cell pellets were incubated at 4°C for 30 min in a lysis buffer (7 M urea, 2 M thiourea, 4% w/v CHAPS, and 10 mM Tris pH 7.4) with proteinase inhibitor cocktail (Roche). The protein concentration was measured with BCA Protein Assay Kit (Thermo, Rockford, IL).

### Western blot analysis

Protein extracts were electrophoresed on 4–12% Bis-Tris gel, transferred to nitrocellulose membranes (Invitrogen), and blocked for 1 h at room temperature in Blocking One (Nacalai tesque, Kyoto, Japan). The membranes were washed three times with 0.1% Tween 20 in Tris-buffered saline and incubated with 2 µg/ml affinity-purified rabbit anti-human ECT2 polyclonal antibody (Santa Cruz Biotechnology, Santa Cruz, CA) overnight at 4°C. The membranes were washed again and incubated with a 1∶10,000 of goat anti-rabbit IgG (H+L) HRP conjugate (Promega, Madison, WI) as a secondary antibody for 2 h at room temperature. Finally, the membranes were detected using SuperSignal West Pico Chemiluminescent substrate (Thermo) and immunoblotting was visualized by exposing the membranes to ATTO Light-Capture II (ATTO, Tokyo, Japan). Signal intensities were quantitated using the CS Analyzer version 3.0 software (ATTO).

### Transfection

OSCC cell lines (Sa3 and H1) were stably transfected with the ECT2 shRNA (shECT2) or the control shRNA (Mock) (Santa Cruz Biotechnology) construct by Lipofectamine LTX and Plus Reagents (Invitrogen). After transfection, the cells stably shECT2 were isolated by the culture medium containing 2 µg/mL puromycin (Invitrogen). 2–3 weeks after transfection, viable colonies were picked up and transferred to new dishes. shECT2- and Mock-transfected cells were used for further experiments.

### IHC

IHC of 4-µm sections of paraffin-embedded specimens was performed using rabbit anti-ECT2 polyclonal antibody (Santa Cruz Biotechnology). Briefly, after deparaffinization and hydration, the endogenous peroxidase activity was quenched by 30-min incubation in a mixture of 0.3% hydrogen peroxide solution in 100% methanol, after which the sections were blocked for 2 h at room temperature with 1.5% blocking serum (Santa Cruz Biotechnology) in PBS before reaction with anti-ECT2 antibody (1∶100 dilution) at 4°C in a moist chamber overnight. Upon incubation with the primary antibody, the specimens were washed three times in PBS and treated with Envision reagent (DAKO, Carpinteria, CA) followed by color development in 3,3′-diaminobenzidine tetrahydrochloride (DAKO). The slides then were lightly counterstained with hematoxylin, dehydrated with ethanol, cleaned with xylene, and mounted. Non-specific binding of an antibody to proteins other than the antigen sometimes occurred. To avoid non-specific binding, an immunizing peptide blocking experiment was performed. As a negative control, triplicate sections were immunostained without exposure to primary antibodies, which confirmed the staining specificity. To quantify the state of ECT2 protein expression in those components, we used IHC score systems described previously [6], [39], [40], [41], [42], [43], [44], [45], [46], [47]. Briefly, the stained cells were determined in at least five random fields at 400× magnification in each section. The intensity of the ECT2 immunoreaction in the cell was scored as follows: 1+, weak; 2+, moderate; and 3+, intense. The cell number and the staining intensity then were multiplied to produce an ECT2 IHC score. Cases with a score exceeding 85.33 (the highest score for normal tissue) were defined as ECT2-positive. Two independent pathologists, both of whom were masked to the patients' clinical status, made these judgments.

### Cellular proliferation

To investigate the effect of shECT2 on cellular proliferation, shECT2- and Mock-transfected cells were seeded in 6-well plates at a density of 1×10^4^ viable cells per well. At the indicated time points, the cells were trypsinized and counted using a hemocytometer in triplicate.

### Cell cycle analysis

To determine cell cycle distribution, the cells were harvested, washed with PBS, and probed with CycleTEST Plus DNA reagent kit (Becton-Dickinson, San Jose, CA), according to the manufacturer's protocol. Briefly, the cells concentrated to 5.0×10^5^ cells/ml were centrifuged at 400×g for 5 min at room temperature. Then we added 250 µl of Solution A (trypsin buffer) to the tube and gently mixed. We allowed the trypsin to react for 10 min at room temperature. Next, we added 200 µl of Solution B (trypsin inhibitor and RNase in a buffer) and gently mix. We incubated with the mixture for 10 min at room temperature. Finally, we added 200 µl of Solution C (propidium iodide stain solution). And we gently mixed as above and incubate for 10 min in the dark on ice. Flow cytometric determination of DNA content was analyzed by a FACScalibur (Becton-Dickinson). The fractions of the cells in the G0–G1, S, and G2–M phases were analyzed using Flow Jo software (Tree Star, Ashland, OR).

### Statistical analysis

The statistical significance of the ECT2 expression levels was evaluated using Fisher's exact test or Mann-Whitney *U* test. *P*<0.05 was considered statistically significant. The data are expressed as the mean ± SEM.
